# Stabilizing Nanoemulsions with Blended Biosurfactants: Role of Sophorolipids and Lecithin in Emulsion Performance

**DOI:** 10.3390/biotech15030051

**Published:** 2026-07-02

**Authors:** Yew Seng Leow, Dayang Radiah Awang Biak, Nur Syakina Jamali, Huey Fang Teh, Norhafizah Abdullah

**Affiliations:** 1 Department of Chemical and Environmental Engineering, Universiti Putra Malaysia, Serdang 43400, Selangor, Malaysia; 2 SD Guthrie Plantation Technology Centre, UPM-MTDC Technology Centre III, Serdang 43400, Selangor, Malaysia; teh.huey.fang@simedarbyplantation.com

**Keywords:** sophorolipids, nanoemulsions, emulsion stability, soybean lecithin

## Abstract

Sophorolipids (SLs) produced from *Starmerella bombicola* using four different secondary substrates such as refined, bleached, and deodorized palm olein (RBD PO), RBD palm kernel olein (RBD PKO), RBD coconut olein (RBD CO) and fatty acid methyl ester (FAME) waste are reported. Their interfacial characteristics at medium-chain triglyceride (MCT) oil-water interface and ability to form nano/submicron emulsions were studied. The effects of SLs from different sources, SL concentrations and blend ratios of SLs and soybean lecithin on characteristics of emulsions produced by ultrasonication were examined. Initially, emulsion formed using SLs coded (from F2 to F5) showed large droplets (d_32_ > 1000 nm) and poor stability. They were then blended with soybean lecithin at a ratio of 3:1 to produce emulsions coded F6 to F9 with smaller droplets (d_32_ < 400 nm) and great stability over a range of temperatures (from 40 °C to 90 °C) and pH values (from 3 to 9). However, highly acidic (pH 2) and low ionic strength (1 mM NaCl) processing caused the separation of the emulsions. These emulsions also displayed potential antimicrobial activities towards *Bacillus cereus* and *Pseudomonas aeruginosa*, as well as cytotoxic effects against the human epithelial colorectal adenocarcinoma cell line (Caco-2). These results illustrated that stable emulsions required a mixture of SLs and soybean lecithin.

## 1. Introduction

Nano- or submicron emulsions are kinetically stable but thermodynamically unstable systems composed of two immiscible liquids. They can be transparent or translucent with droplet sizes of 50–200 nm (nanoemulsions) or milky with droplet sizes up to 500 nm (submicron emulsions) [1]. Surfactants are usually used to reduce the interfacial tension which leads to the improvement of droplet dispersion and emulsification [2]. Recently, attention has been focused on natural surfactants due to a stronger preference of natural ingredients over synthetic additives in commercial products, which creates a ‘clean-label’ trend among manufacturers [3].

One commonly used natural surfactant is lecithin, which can be extracted from plant-based oils or animal tissues. Even among other sources, the soybean lecithin is the most preferred of lecithin due to its availability, low toxicity, and high nutritional value. Its high content of phospholipids and triglycerides enables it to display excellent emulsifying and surface-wetting ability and high biocompatibility with other bioactive compounds [4]. However, Rocchio et al. [5] stated that soybean lecithin cannot form stable emulsions when it is used as the only surfactant due to the absence of electrostatic repulsion between oil droplets.

Sophorolipids (SLs) are extracellular glycolipid biosurfactants produced by yeasts such as *Rhodotorula bogoriensis*, *Candida kuoi*, *Candida tropicalis*, *Candida bombicola*, and *Wickerhamiella domercqiae* [6]. They consist of a hydrophilic sophorose moeity (diglucose with a β-1,2 bond) attached to a hydrophobic part of hydroxylated fatty acid (mainly C18:1) via a glycosidic bond. SLs are typically produced as crude mixtures of acidic and lactonic forms, which strongly influence their interfacial, hydrophilic, and antimicrobial properties. Previous studies have applied SLs in emulsion formation using homogenization and high SL concentrations [7,8]; however, these systems often resulted in large droplet sizes that increased over time. Combining SLs with phospholipids such as lecithin has been shown to reduce droplet size, enhance micelle formation, and improve emulsion stability, offering advantages over single-surfactant systems.

In this study, oil-in-water nanoemulsions were prepared using SLs produced by *S. bombicola* from four different secondary substrates—RBD PO, RBD PKO, RBD CO, and FAME waste—and their interactions with lecithin were evaluated. The effects of environmental stresses, including centrifugation, temperature, pH, and storage, on nanoemulsion stability were assessed, along with creaming, aggregation, antimicrobial activity, and cytotoxicity. This work provides insights into how substrate composition and biosurfactant–lecithin synergy influence nanoemulsion properties, stability, and functional performance, advancing the development of SL-based delivery systems for biotechnological applications.

## 2. Materials and Methods

### 2.1. Chemical

Yeast Malt (YM) broth (Bio Basic, Markham, ON, Canada) was used to cultivate *S. bombicola*. For the production of SLs, basal growth media with the composition of 100 g L^−1^ D-glucose (R&M Chemicals, Puchong, Malaysia), 1.0 g L^−1^ magnesium sulphate heptahydrate (R&M Chemicals, Kingston upon Hull, UK), 5.0 g L^−1^ monopotassium hydrogen phosphate (Bio Basic, Markham, ON, Canada), 0.1 g L^−1^ sodium chloride (Bio Basic, Markham, ON, Canada), 0.1 g L^−1^ calcium chloride (Bio Basic, Markham, ON, Canada), and 5.0 g L^−1^ yeast extract (Fischer BioReagents, Pittsburgh, PA, USA) were used. RBD PO and RBD CO were procured from a local market in Seri Kembangan, Selangor, and Ayam Brands, Malaysia, respectively, while RBD PKO were provided by the Sime Darby Plantation Technology Centre, UPM. Meanwhile, FAME waste was obtained from Sime Darby Oils Sdn. Bhd., Malaysia. For extraction and recovery of the SLs, two analytical-grade solvents, such as hexane and ethyl acetate (R&M Chemicals, Puchong, Malaysia), were utilized.

Medium-chain triglycerides (MCT, 70:30) (Oleon Asia-Pacific, Klang, Malaysia) and soybean lecithin BolecTM ZT, Sime Darby Oils (Zwijndrecht, The Netherlands) were used in the nanoemulsion preparation.

### 2.2. Microbial Culture and Production of Sophorolipids

*S. bombicola* (DSM 27465) inoculum was obtained by transferring 20 colonies grown on yeast malt agar into 100 mL of sterile growth medium with a pH 5.5 of in a 250 mL Erlenmeyer flask. The culture was incubated at 30 °C with a mixing speed of 210 rpm for 20 h in an orbital shaking incubator (New Brunswick Scientific, Enfield, CT, USA). It was then harvested by centrifugation at 2200× *g* for 10 min (Eppendorf, Hamburg, Germany). The cell pellet was resuspended with a fresh and sterile medium. An inoculum of 5% (*v*/*v*) of the seed culture prepared previously was transferred into a batch shake flask culture of 250 mL containing a sterile growth medium supplemented with different secondary carbon substrates (RBD PO, RBD PKO, RBD CO, and FAME waste) at 10% (*v*/*v*). These cultures were then cultivated for 8 days at 30 °C and 210 rpm in a shaker incubator (New Brunswick Scientific, Edison, NJ, USA).

### 2.3. Recovery of Sophorolipids

Extraction of the SLs was performed according to the method described by Shah et al. [9] with some modification. Briefly, the top layer containing residual oil was removed with n-hexane. The bottom layer containing the fermentation broth, cell pellet, and SLs was centrifuged at 10,000× *g* for 15 min to remove the medium. Then, the yeast biomass and SLs were resuspended in deionised water. An equivalent volume of ethyl acetate was added into the solution and shaken vigorously using vortex for 5 min. The ethyl acetate phase was taken and later vacuum dried at 40 °C using a centrifugal evaporator EZ-2 (Genevac, Ipswich, Suffolk, UK). The SLs recovered were stored at 4 °C in a chiller for further studies.

### 2.4. Fatty Acid Compositions of Sophorolipids

The SLs produced in this study were studied for their fatty acid compositions via gas chromatography-flame ionization detection (GC-FID, Agilent Technologies, Santa Clara, CA, USA) according to the procedures stated by Morya et al. [10] with modifications. Briefly, the sample mass was fixed at 0.05 g, and saponification was carried out using 0.5 M KOH in methanol at 70 °C. Fatty acid methyl esters were prepared using 14.0% (*w*/*v*) boron trifluoride–methanol. Following methylation, hexane extraction and the addition of saturated NaCl solution were employed to enhance phase separation. GC-FID operating conditions were modified, including the use of a Supelcowax 10 column (30 m × 0.53 mm × 1 µm), a temperature programme of 170–230 °C with a heating rate of 5 °C min^−1^, and injector and detector temperatures set at 250 °C and 300 °C, respectively.

### 2.5. Emulsion Preparation

Emulsion was prepared according to the method used by Ghosh et al. [11]. A 10% (*v*/*v*) oil phase (medium-chain triglycerides) was mixed with 90% (*v*/*v*) aqueous phase containing 1.0 wt% of different biosurfactants. Emulsions were produced by adding drops of oil into the aqueous phase while mixing it with a homogenizer (IKA, T25 Ultra Turrax, Staufen im Breisgau, Germany), followed by ultrasonication (Sonics, Vibra-Cell VCX 130, Newtown, CT, USA) at 60% amplitude for 10 min.

### 2.6. Characteristics of Emulsions

#### 2.6.1. Particle Size

Emulsion samples were added dropwise into the wet dispersion unit (Hydro MV, Malvern, Worcestershire, UK). The droplet diameter of each sample was then measured and recorded as a surface-weighted mean diameter (d_32_) using a laser diffraction instrument (Malvern, Mastersizer 3000, Worcestershire, UK). The refractive index (RI) of water and MCT oil with values of 1.330 and 1.445, respectively, was used as a baseline reference.

#### 2.6.2. Viscosity

A spindle coded ELVAS-SP attached to a viscometer (IKA, Staufen im Breisgau, Germany) at a speed of 45 rpm at 25 °C was used for viscosity measurement.

#### 2.6.3. Stability Test

A total of 5 mL of emulsion was placed into a 15 mL test tube and centrifuged for 30 min at 3500 rpm and at 25 °C (Eppendorf, Hamburg). No phase separation formation was observed [12].

#### 2.6.4. Measurement of Zeta Potential

Zeta potential value, or droplet charge was quantified using a Zetasizer (Malvern, Zetasizer Nano-ZS, Worcestershire, UK). To avoid numerous scattering effects, the samples were diluted with deionized water (1:100, *v*/*v*) before measurement.

#### 2.6.5. Antimicrobial Assay

The agar well diffusion method was applied to screen antimicrobial activities of different emulsion formulations [13]. Two pathogenic strains, *Bacillus cereus* and *Pseudomonas aeruginosa*, kindly provided by Sime Darby Plantation Technology Centre, were used in this study. Both strains were initially streaked onto nutrient agar (Oxoid, Hampshire, UK) and incubated overnight at 30 °C and 37 °C, respectively. A single colony from each plate was then resuspended in 1 mL of sterile distilled water and spread onto fresh nutrient agar plates. Wells of 6.5 mm diameter were created in the agar using a sterile cork borer, and 100 µL of SL suspensions (5 mg/mL in deionized water) were added to each well. Plates were incubated for 16 h at 30 °C for *B. cereus* and 37 °C for *P. aeruginosa.* Antimicrobial activity was assessed by measuring the diameter of the inhibition zones, including the well, after the incubation period.

#### 2.6.6. Cytotoxicity Assay

The cytotoxicity assay using MTT (3-[4,5-dimethylthiazol-2-yl]-2,5 diphenyltetrazolium bromide) was based on the method described by Yoon et al. [14]. Two human cell lines chosen for this test were human lung fibroblasts, MRC5, and human epithelial colorectal adenocarcinoma cell lines, Caco-2, purchased from the American Type Culture Collection (Manassas, VA, USA). The diluted nanoemulsion ranges of 500 µg, 250 µg, 125 µg, 75 µg, 25 µg, 10 µg, and 1 µg mL^−1^ were applied to each well and incubated for 72 h. The MTT solution was later applied to the cells, followed by incubation for 3 h. The Optical Density (OD) of the emulsion was measured using an ELISA reader (Tecan Sunrise, Zürich, Switzerland) at a wavelength of 570 nm after the purple formazan crystals were solubilized with DMSO. The cell viability was calculated using Equation (1) below:

(1) $$Cell\;viability\left(\%\right)=\frac{A_{sample}-A_{blank}}{A_{control}-A_{blank}}\times 100\%$$

where

*A_sample_*: Absorbance of treated cells with nanoemulsions
*A_control_*: Absorbance of untreated cells
*A_blank_*: Absorbance of blank (medium without cells)

Using the values for calculated percentage of cell viability from Equation (1), graphs were plotted with cell viability (%) versus different concentrations of stable emulsions.

### 2.7. Nanoemulsions Stability Testing

The aim of this test is to determine how the effect of various environmental circumstances on the stability of nanoemulsions such as flocculation, Ostwald ripening, gravitational separation, and coalescence may contribute to emulsion instability. Freshly prepared nanoemulsion formulations were exposed to various environmental conditions and later stored for 24 h at room temperature. Then, their stability was determined through physical observation and change in average particle size, as described by Bai and McClements [15].

#### 2.7.1. Stability Under Thermal Environment

The stable nanoemulsion formulations were placed in the glass test tubes and subjected to water baths of different temperatures ranging from 30 °C to 90 °C for 30 min.

#### 2.7.2. Stability Under Different pH

Stable nanoemulsions formulations were placed in 10 mL beakers, which were then adjusted to various pH values ranging from 2.0 to 9.0 using NaOH or HCl solutions before being transferred to glass test tubes.

#### 2.7.3. Stability Under Different Ionic Strength

Stable nanoemulsion formulations were placed in 10 mL beakers. They were then adjusted to various salt concentrations ranging from 1 mM to 300 mM NaCl by adding concentrated NaCl solution followed by transferring into glass test tubes.

#### 2.7.4. Accelerated Centrifugal Stability

A 5 mL aliquot of each nanoemulsion formulation was transferred into a 15 mL centrifuge tube and centrifuged at 3500 rpm for 30 min at 25 °C using a Centrifuge 5810 R (Eppendorf, Hamburg, Germany). Following centrifugation, the samples were visually inspected for any signs of phase separation. Formulations that showed no observable phase separation were classified as stable emulsions.

#### 2.7.5. Storage Test

The stable nanoemulsion formulations were evaluated for a month at various storage temperatures of 4 °C, 25 °C, and 40 °C [16]. The particle size was monitored every five days using a laser diffraction instrument (Malvern, Mastersizer 3000, Worcestershire, UK).

### 2.8. Interfacial Tension Measurement

For this test, an MCT oil-water interfacial tension was examined at 25 °C using a tensiometer (Dataphysics, Stuttgart, Germany) and a Wilhelmy platinum plate as a probe for measurement. The biosurfactant concentration used in the experiment ranged from 0.1 to 2.0 (wt%). Interfacial tension (IFT) values obtained were expressed in the unit of mN/m and displayed as the function of the biosurfactant concentration.

### 2.9. Critical Micelle Concentration (CMC)

CMC is defined as the surfactant concentration at which micelles begin to form [17]. To determine the CMC of the SLs, surface tension was measured for concentrations ranging from 0 mg/L to 200 mg/L. All measurements were performed in triplicate, and results are presented as mean ± SD. The CMC was obtained by plotting the surface tension against the concentration and identifying the intersection of two linear fits representing the pre- and post-micelle formation regions.

### 2.10. Statistical Analysis

All measurements were performed using three independently and freshly prepared samples. For each experimental variable, the data were subjected to statistical evaluation and are presented as mean ± standard deviation (SD). Triplicate analyses were used to ensure the reproducibility and reliability indication of the samples’ performance.

## 3. Results

### 3.1. Chemical Compositional of SLs

GC–FID analysis was conducted to determine the fatty acid composition of the SLs produced. This analysis was essential because different secondary substrates, each containing distinct fatty acid profiles, were supplemented during the fermentation of *S. bombicola.* In the GC–FID procedure, fatty acids in the SLs were first esterified to form fatty acid esters, which were then detected and quantified, as presented in Table 1.

The SLs derived from RBD PO were dominated by palmitic acid (C16:0) and oleic acid (C18:1), with abundances of approximately 34.86% and 45.94%, respectively. This finding is consistent with the fatty acid composition of the RBD PO substrates. For SLs obtained from the RBD PKO, the predominant fatty acids were lauric acid (C12:0, 41%), oleic acid (C18:1, 22%), and myristic acid (C14, 13%). Similarly, SLs produced from RBD CO were rich in medium-chain fatty acids, particularly lauric acid (47%) and myristic acid (17%). These results align with the GC–FID profiles of RBD PKO and RBD CO, where high levels of medium-chain fatty acids (C12 and C14) were observed. Interestingly, for SLs produced from FAME waste, several fatty acids—including C12, C14, C16:1, and C18:2—were detected in the SLs despite not being present in the original substrate. This suggests that the complex mixture of compounds in FAME waste may have enabled *S. bombicola* to synthesize a broader range of fatty acid moieties during SL production.

Overall, the GC–FID analysis clearly demonstrates that the fatty acid moiety of sophorolipids is strongly influenced by the type and composition of the fatty acids supplied to *S. bombicola* during fermentation.

### 3.2. Interfacial Tension of Biosurfactant

The SLs extracted from RBD PO, RBD PKO, RBD CO, and FAME waste, as well as soybean lecithin, were studied for their IFT and CMC values between deionized water and MCT oil using the Wilhelmy plate method. Table 2 shows the IFT and CMC values for SLs, soybean lecithin, and mixtures of SLs and soybean lecithin. For all SLs, the IFT values were around 5 and 5.5 mN m^−1^ with CMC values ranging from 0.52 to 0.7 wt%. Soybean lecithin showed IFT and CMC values of 1.50 ± 0.31 mN m^−1^ and 0.90 wt%, respectively. Meanwhile, lower IFT values ranging from 3.75 ± 0.10 to 4.47 ± 0.11 mN m^−1^ for mixtures of SLs and soybean lecithin, but higher CMC values of 1.0 wt% were determined.

### 3.3. Formation of Emulsions Stabilized by Sophorolipids and Soybean Lecithin

For this study, SLs and soybean lecithin were added separately into aqueous phase containing deionised water while MCT oil was used as the oil phase. They were mixed using a homogenizer, followed by ultrasonication to form emulsion, which would be studied for their stability and properties.

#### 3.3.1. Effect of Biosurfactant Concentration on Emulsion Formation

**Figure 1:**
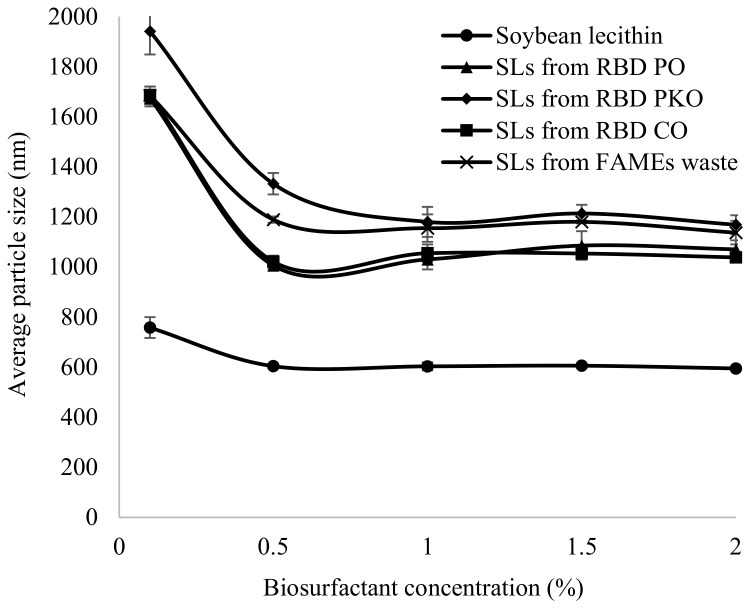
Effect of a biosurfactant concentration using individual SLs or soybean lecithin on the average particle size of an emulsion.

Figure 1 displayed the influence of different concentrations of biosurfactants on an average particle size of emulsion. The ability of SLs to emulsify was compared to that of soybean lecithin. There was a two-regime profile observed for emulsions prepared using SLs or soybean lecithin, with the initial decrease in average particle size when the surfactant concentration increased from 0.1 wt% to 0.5 wt% preceded by a relatively constant particle size at a high concentration of surfactant (from 0.5 wt% to 2.0 wt%). The emulsion with SLs from RBD PKO exhibited the largest particle size of 1180 ± 60 nm followed by SLs from FAME waste, RBD CO, and RBD PO with particle sizes of 1155 ± 55, 1055 ± 35 nm and 1030 ± 40 nm, respectively. Meanwhile, the emulsion produced using soybean lecithin has a smaller droplet size of 603 ± 16 nm.

**Figure 2:**
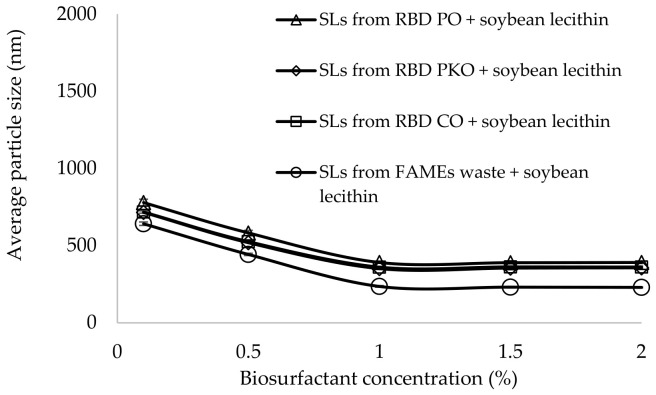
Effect of a biosurfactant concentration using the SLs-soybean lecithin mixture on the average particle size of the emulsion.

The effect of blending two biosurfactants was evaluated by mixing soybean lecithin with SLs from different origins, namely RBD PO, RBD PKO, RBD CO, and FAME waste at a 1:3 ratio. As shown in Figure 2, the smallest particle size of 229 ± 3 nm was observed for F9, followed by F7, F8, and F6, with particle sizes of 356 ± 5 nm, 361 ± 6, and 391 ± 7 nm, respectively, at a total biosurfactant concentration of 2 wt%.

#### 3.3.2. Effect of Soybean Lecithin to Sophorolipids Ratio on Formation of Emulsion

**Figure 3:**
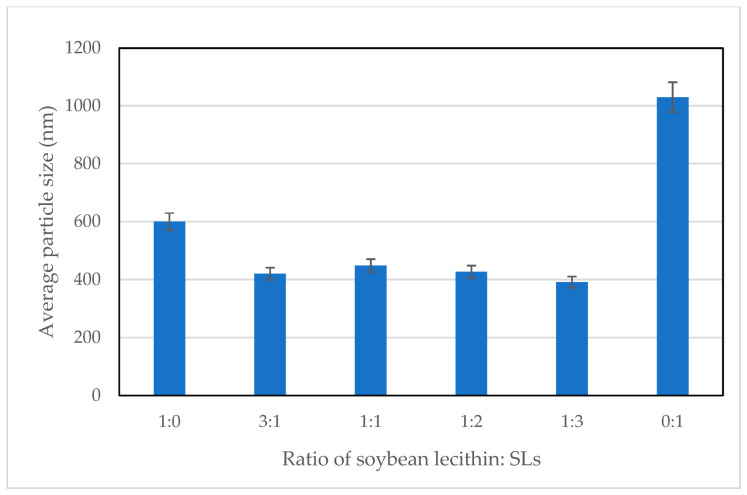
Effect of soybean lecithin to SL ratio on the average particle size of emulsion produced with SLs from RBD PO.

For this study, SLs from RBD PO were selected to investigate the effect of different soybean lecithin to SL ratios on the emulsion particle size. Emulsions were prepared using ratios of 1:0, 1:1, 1:2, 1:3, and 0:1 at a final surfactant concentration of 1.0 wt%. As shown in Figure 3, the average particle size decreased dramatically from 1030 ± 40 nm to 391 ± 5 nm at a 1:3 ratio, corresponding to 0.25 wt% soybean lecithin and 0.75 wt% SLs. Particle size then increased to 427 ± 12, 448 ± 14, and 452 ± 17 nm for ratios of 1:2, 1:1, and 3:1, respectively.

#### 3.3.3. Formulation Blends of Emulsions

The formulation blends of the emulsion with their respective components were examined for their viscosity, particle size, centrifugation stability, and zeta potential. All emulsions had a milky appearance (Table 3).

Emulsions coded F1, F6, F7, F8, and F9 had greater viscosity of 1.64 ± 0.01, 1.70 ± 0.02, 1.64 ± 0.02 mPa.s, 1.54 ± 0.02 mPa.s, and 1.58 ± 0.02 mPa.s, respectively. These values were higher than emulsions coded F2, F3, F4, and F5 with viscosities of 1.34 ± 0.04, 1.21 ± 0.02, 1.30 ± 0.02, and 1.36 ± 0.02, respectively. The centrifugation stability test was also performed to determine the stability of an emulsion using a high mechanical stress, which was comparable to the gravitational forces for one year [12]. Stable formulation blends were F1, F6, F7, F8, and F9, while unstable emulsions were F2, F3, F4, and F5, which showed phase separation after being centrifuged. Thus, F6 to F9 showed that the emulsions with higher viscosity and smaller droplet size were more stable.

Next, the zeta potentials of F1, F6, F7, F8, and F9 were assessed with the highest value of −80 ± 2.5 mV, followed by F6, F8, F7, and F9 with −75.2 ± 1.4 mV, −69.5 ± 0.2 mV, −63.4 ± 0.8 mV, and −61.7 ± 0.2 mV, respectively. As compared to F2, F3, F4, and F5, with only SLs, their zeta potential values between +30 mV and −30 mV (not measured due to separation after centrifugation stability test) indicated that the addition of soybean lecithin increased the zeta potential and stability of the emulsions coded F6 to F9.

The polydispersity index (PDI) values of the formulations are summarized in Table 3 as well. Emulsion blend F1 exhibited a PDI of 0.567, whereas F9 showed a markedly lower value of 0.04. Formulations F6, F7, and F8 had similar PDI values of 0.235, 0.212, and 0.236, respectively. These results indicate differences in particle size distribution among the emulsion blends.

As a result of this study, stable formulation blends F1, F6, F7, F8, and F9 were chosen and put to the test for further investigation.

### 3.4. Influence of Environmental Stresses on Nanoemulsion Stability

Emulsion-based products were required to retain their physical and chemical stability under different environmental pressures because gravitational separation, flocculation, coalescence, and Ostwald ripening will influence the bioactive molecules in the emulsion. F1, F6, F7, F8, and F9 were chosen to assess their stability by exposing them to rigorous environmental conditions such as thermal, pH, ionic strength, and storage stability.

#### 3.4.1. Thermal Stability

**Figure 4:**
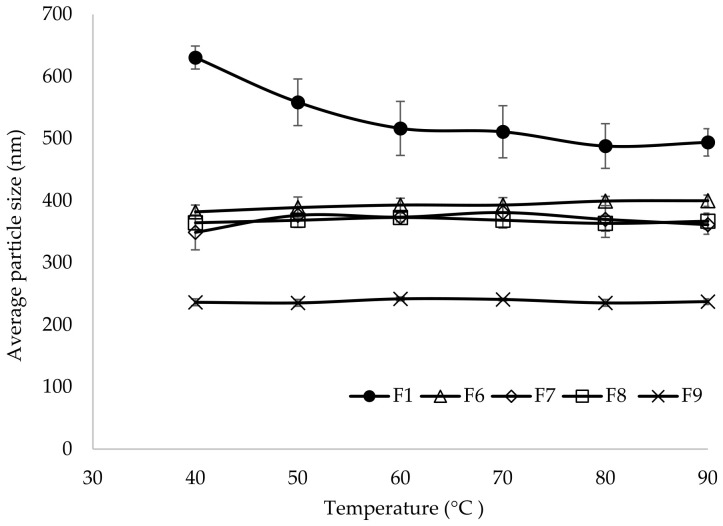
Effect of thermal processing (40–90 °C) on the particle size of nanoemulsions formulation blends.

The thermal stability of F1, F6, F7, F8, and F9 nanoemulsions was investigated by immersing them in water baths at temperatures ranging from 40 °C to 90 °C for 30 min, followed by cooling to room temperature for 24 h. Figure 4 depicts that the nanoemulsions F6, F7, F8, and F9 were thermally stable at the various temperatures (from 40 °C to 90 °C) examined, as evidenced by a lack of significant changes in the average particle size as well as no phase separations. As for the F1 blend, a reduction in the average particle size with temperature was observed.

#### 3.4.2. pH Stability

**Figure 5:**
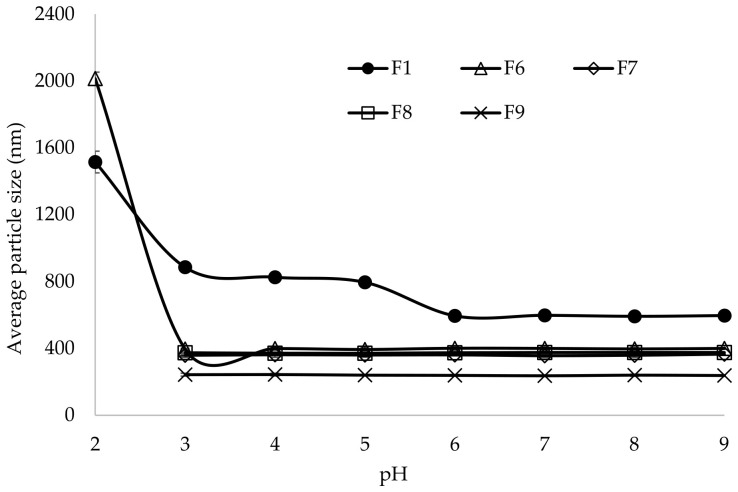
Effect of pH stress (pH 2 to 9) on the particle size of nanoemulsions formulation blends.

In Figure 5, the average droplet diameter and physical stability of nanoemulsions were assessed across a pH range of 2 to 9. When exposed to pH 3 to 9, F6, F7, F8, and F9 remained resistant to creaming and oiling off and exhibited no change in the average particle size. In contrast, the average particle size in F1 began to increase between pH 3 and pH 5. Interestingly, all formulations showed a sharp increase in average particle size when exposed to pH 2.

#### 3.4.3. Ionic Strength

**Figure 6:**
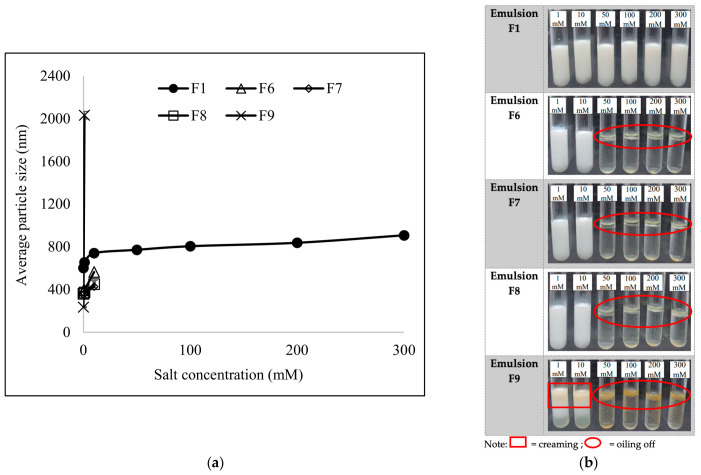
(**a**) Effect of ionic strength on the particle size of nanoemulsions formulation blends; (**b**) visual observation of emulsion blends at different ionic strengths.

For this study, different concentrations of salt (0–300 mM) were applied to investigate the influence of ionic strength on the stability of nanoemulsions as shown in Figure 6. The increase in salt concentration from 0 mM to 300 mM caused the average particle size to grow steadily from 603 ± 16 nm to 908 ± 13 nm for F1. In contrast, the average particle size increased to 565 ± 36, 428 ± 44, and 450.5 ± 33.5 nm for formulations F6, F7, and F8, respectively. Formulation F9 showed the largest increase in average particle size from 236 ± 5 nm to 2032.5 ± 35 nm when evaluated at a low salt concentration of 1 mM. Phase separation was detected in formulations F6, F7, F8, and F9 when the salt concentration rose from 50 mM to 300 mM.

#### 3.4.4. Storage Stability

The stability of 10% MCT nanoemulsions (F1, F6, F7, F8, and F9) was assessed over one month at 4 °C, 25 °C, and 40 °C following the method of Shu et al. [16]. All formulations were tested at pH 7.0 without added salt. No significant changes in mean particle size were observed for the F6–F9 samples stored at 4 °C and 25 °C. However, a slight increase in droplet size was noted for F1, from 603 ± 16 nm to 700 ± 29 nm after 30 days at 25 °C. At an elevated temperature of 40 °C, only F6 and F9 maintained their particle sizes after one month, with final droplet diameters of 406 ± 19 nm and 251 ± 3 nm, respectively. The stability of the F1 emulsion lasted only five days, while F7 and F8 were stable up to ten days at 40 °C. After 30 days, the droplet sizes of F1, F7, and F8 had increased to 1236 ± 23 nm, 700 ± 14 nm, and 516 ± 9 nm, respectively.

#### 3.4.5. Interfacial Characteristic of SLs

SLs from RBD PO, RBD PKO, RBD CO, and FAME waste were tested for their interfacial properties using deionised water and MCT oil as the hydrophilic and hydrophobic phases, respectively. The effect of different SL concentrations (0–2 wt%) on interfacial tension (IFT) was measured using the Wilhelmy plate method. The initial IFT between deionised water and MCT oil without SLs was 24.00 ± 0.42 mN/m. As SL concentrations increased to 2 wt%, IFT decreased to a constant range of 4.6–5.6 mN/m. At 2 wt%, the order of IFT for SLs from different sources was: FAME waste (4.65 ± 0.14 mN/m), RBD PO (5.07 ± 0.33 mN/m), RBD CO (5.43 ± 0.14 mN/m), and RBD PKO (5.52 ± 0.26 mN/m).

CMC values obtained were 0.52 wt% for SLs from FAME waste, 0.6 wt% for SLs from RBD PO and RBD CO, and 0.7 wt% for SLs from RBD PKO. SLs effectively reduced IFT even at low concentrations (0.1 wt%), with IFT dropping from 24 mN/m to 8–12 mN/m, indicating micelle formation.

Soybean lecithin was also tested for IFT and CMC as it was blended with SLs for nanoemulsion formulation. The CMC of soybean lecithin alone was 0.9 wt%, and it reduced IFT from 24.00 ± 0.42 mN/m to 1.50 ± 0.31 mN/m. When SLs were blended with soybean lecithin, the mixture at 0.1 wt% already reduced IFT more effectively (8–10 mN/m) than SLs alone (8.5–12 mN/m). As the concentration increased to 2 wt%, IFT values for SLs-soybean lecithin mixtures decreased further to 3.75–4.47 mN/m, with CMC slightly higher at 1.0 wt%.

### 3.5. Antimicrobial and Cytotoxicity Assessment of Nanoemulsions Formulation Blends

Nanoemulsions formulations (F1, F6, F7, F8, and F9) were tested for their antimicrobial properties against Gram-positive and Gram-negative bacteria, which were *B. cereus* and *P. aeruginosa*, respectively. These strains were selected given the growing interest in enhancing food products through nanotechnology, emphasizing their potential application as nutraceuticals.

Table 4 lists the zones of inhibition for nanoemulsions against *B. cereus* and *P. aeruginosa*. Inhibition against *B. cereus* was observed by all formulations except F1. F9 displayed the highest inhibition zone of 25.4 ± 0.5 mm, followed by F6, F7, and F8, with values of 14.2 ± 0.6 mm, 14.2 ± 0.7 mm, and 13.5 ± 0.4 mm, respectively. In contrast, all nanoemulsions exhibited lower antimicrobial activity against *P. aeruginosa* with inhibition zones of ~7.4 mm.

Cytotoxicity assay was performed to assess the cytotoxic potential of nanoemulsions formulations against human lung fibroblast (MRC5) and human epithelial colorectal cancer (Caco-2) cell lines. For the MRC5 cell line, only the F6 formulation showed cytotoxicity activity with IC_50_ at 420 μg/ mL (Table 4). Meanwhile, for the Caco-2 cell line, the lowest IC_50_ value of (35 μg/ mL) was observed for F1. Formulations F7 and F8 showed moderate cytotoxicity with IC_50_ values of 65 μg/mL and 70 μg/mL, respectively, whereas F6 and F9 required higher concentrations (130 μg/mL and 85 μg/mL, respectively) to achieve 50% inhibition of the Caco-2 cells.

## 4. Discussion

The formation, stability, and functional properties of the nanoemulsions were strongly influenced by surfactant type, concentration, ratio, and environmental conditions. Emulsions stabilized solely with sophorolipids (SLs) exhibited relatively large droplet sizes, consistent with previous reports that SLs alone form emulsions with droplet sizes exceeding 1000 nm due to limited interfacial coverage and osmotic attraction between micelles and oil droplets [7,8,18]. In contrast, emulsions containing soybean lecithin produced smaller droplets, reflecting rapid adsorption of phospholipids at the oil-water interface, which efficiently encapsulates oil droplets and enhances surface activity [19].

Blending SLs with soybean lecithin further reduced droplet size and enhanced emulsion stability. The smallest droplets were obtained at a soybean lecithin to SLs ratio of 1:3, indicating an optimal balance that maximizes interfacial adsorption without saturating the interfacial layer, which could otherwise reduce synergistic effects [20,21]. The polydispersity index (PDI) values supported these observations: F1 exhibited a relatively high PDI of 0.567, indicating a broad droplet size distribution, whereas F6, F7, and F8 had moderate PDI values of 0.235, 0.212, and 0.236, reflecting improved uniformity. F9 displayed an exceptionally low PDI of 0.04, demonstrating highly monodisperse droplets. Formulations F1, F6, F7, F8, and F9, which combined higher viscosities, smaller droplet sizes (<400 nm), and low PDI values, also exhibited superior centrifugation stability and high negative zeta potentials (from −61.7 mV to −80 mV), indicating strong electrostatic repulsion and colloidal stability [22]. These characteristics confirm that the combination of SLs and soybean lecithin produces thicker interfacial layers that resist Ostwald ripening and coalescence, classifying the emulsions as nanoemulsions (<500 nm) [1].

The lowest interfacial tension (IFT) was observed for SLs derived from FAME waste; this is likely due to its high C18:1 fatty acid content (≈57%), which facilitates more efficient adsorption at the MCT oil-water interface. In contrast, SLs from RBD PKO and RBD CO exhibited similar IFT values, consistent with their comparable C12 and C14 fatty acid compositions, supporting previous findings that high C14 content increases IFT [23]. The higher critical micelle concentration (CMC) values observed for SLs and SLs-soybean lecithin mixtures compared to modified SLs (e.g., SL-ethyl or SL-hexyl esters) can be attributed to differences in molecular modification and interfacial thickness. While lower CMC values indicate more efficient micelle formation, higher CMC values in the mixtures likely reflect the formation of thicker interfacial layers, which enhances emulsion stability [24].

Blending SLs with soybean lecithin also further reduced the IFT due to the hydrophobic–hydrophobic interactions between the fatty acid moieties of the SLs and the phospholipids in the lecithin. These interactions overcome electrostatic repulsion between negatively charged groups (COOH in SLs and phosphate/carbonyl in lecithin), promoting more effective adsorption at the oil-water interface and enhanced emulsification compared to SLs alone. Overall, the interfacial properties and CMC values indicate that both the source of SLs and their combination with lecithin significantly influence adsorption behaviour, micelle formation, and the formation of stable nanoemulsions.

Environmental stress tests showed that F6–F9 maintained droplet size and resisted phase separation from 40 °C to 90 °C and pH 3. This is attributable to the low molecular weight of SLs interacting with lecithin to form thick interfacial coatings and provide high electrostatic repulsion [15,25,26,27]. Storage studies revealed minimal droplet growth at 4 °C and moderate increases at 25 °C, while higher temperatures (40 °C) accelerated droplet collisions and caused partial destabilization due to emulsifier dehydration [28]. Conversely, increasing ionic strength reduced interfacial tension and weakened electrostatic repulsion, causing droplet aggregation and phase separation [29], indicating that high salt concentrations remain a limitation for nanoemulsion stability.

Nanoemulsion composition also influenced biological activity. F6-F9 exhibited antibacterial activity against Gram-positive (*B. cereus*) and Gram-negative (*P. aeruginosa*) bacteria, with F9 showing the highest inhibition against *B. cereus*, likely due to the smaller droplet size enhancing diffusion into bacterial cells. F1 inhibited Gram-negative bacteria, possibly due to structural similarities between lecithin phospholipids and bacterial membranes [30]. Cytotoxicity assays revealed selective activity toward human colorectal cancer cells (Caco-2), with F1 showing the highest effect (IC_50_ = 35 μg/mL), likely because soybean lecithin facilitates membrane penetration [31]. Formulations F7 and F8, which contained SLs rich in shorter-chain fatty acids (C12 and C14), displayed greater cytotoxicity than F6 and F9 due to the easier release of SLs from oil droplets, which enhanced their interaction with the cancer cells [32].

Overall, these findings highlight that the combination of SLs and soybean lecithin optimizes droplet size, interfacial stability, and resistance to thermal and pH stresses, while modulating biological activity.

## 5. Conclusions

Nanoemulsions formulated with sophorolipids (SLs) and soybean lecithin exhibit enhanced physicochemical stability, reduced droplet size, and improved biological functionality compared to those formulated with SLs alone. The optimal soybean lecithin to SLs ratio of 1:3 produced nanoemulsions with small, uniform droplets (<400 nm), high viscosity, and strong electrostatic repulsion, contributing to their resistance to thermal and pH stresses. While their stability under high ionic strength and elevated temperatures remains a limitation, these formulations maintained long-term storage stability under moderate conditions. The potential of these nanoemulsions to serve as functional nutraceuticals and as platforms for targeted antimicrobial and anticancer applications was also demonstrated.

## Figures and Tables

**Table 1:** The percent abundance of the fatty acids chain in SLs derived from different substrates.

Fatty Acid Chain Length	Source of SLs Substrate			
	RBD PO	RBD PKO	RBD CO	FAME Waste
C8	-	4.16	6.83	-
C10	<1	3.90	6.01	-
C12	<1	41.59	47.45	3.43
C14	<1	13.79	17.91	1.27
C16	34.86	8.23	8.31	18.75
C16:1	<1	<1	<1	10.83
C18	3.75	2.26	2.58	5.08
C18:1	45.94	22.83	8.41	57.22
C18:2	13.21	2.82	1.81	2.56
C18:3	<1	<1	<1	<1
C20	<1	<1	<1	<1
C20:1	<1	-	-	-

Note: - = not detected.

**Table 2:** Interfacial tension (IFT)and critical micelle concentration (CMC) of different biosurfactant blend.

Emulsion Blend	Biosurfactant Formulation	IFT (mN m^−1^)	CMC (wt%)
F1	Soybean lecithin	1.50 ± 0.31	0.90
F2	SLs from RBD PO	5.07 ± 0.33	0.70
F3	SLs from RBD PKO	5.52 ± 0.26	0.60
F4	SLs from RBD CO	5.43 ± 0.14	0.60
F5	SLs from FAME waste	4.65 ± 0.14	0.52
F6	SLs from RBD PO + soybean lecithin	4.29 ± 0.11	1.00
F7	SLs from RBD PKO + soybean lecithin	4.36 ± 0.07	1.00
F8	SLs from RBD CO + soybean lecithin	4.47 ± 0.11	1.00
F9	SLs from FAME waste + soybean lecithin	3.75 ± 0.10	1.00

**Table 3:** Characteristic of different emulsions formulation blends.

Emulsion Blend	Composition	Viscosity (mPa.s)	Particle Size, d_32_ (nm)	Centrifugation Stability Test	Zeta Potential (mV)	Polydispersity Index
F1	1 wt% BL	1.64 ± 0.01	603 ± 16	√	−80.0 ± 2.5	0.567 ± 0.027
F2	1 wt% SLs from RBD PO	1.34 ± 0.04	1030 ± 40	X	*	*
F3	1 wt% SLs from RBD PKO	1.21 ± 0.02	1180 ± 60	X	*	*
F4	1 wt% SLs from RBD CO	1.30 ± 0.02	1055 ± 35	X	*	*
F5	1 wt% SLs from FAME waste	1.36 ± 0.02	1155± 55	X	*	*
F6	0.75 wt% SLs from RBD PO + 0.25 wt% BL	1.70 ± 0.02	391 ± 5	√	−75.2 ± 1.4	0.235 ± 0.001
F7	0.75 wt% SLs from RBD PKO + 0.25 wt% BL	1.64 ± 0.02	351 ± 12	√	63.4 ± 0.8	0.212 ± 0.001
F8	0.75 wt% SLs from RBD CO + 0.25 wt% BL	1.54 ± 0.02	361 ± 4	√	69.5 ± 0.2	0.236 ± 0.001
F9	0.75 wt% SLs from FAME waste + 0.25 wt% BL	1.58 ± 0.02	236 ± 5	√	−61.7 ± 0.2	0.094 ± 0.004

Note: BL = soybean lecithin; SLs = sophorolipids; * = zeta potential, PDI not conducted as the formulation failed their respective stability analysis; √ = pass with no phase separation; X = failed (phase separation occurred).

**Table 4:** Antimicrobial and Cytotoxicity properties of different emulsions formulation blends.

Emulsion Blend	Antimicrobial Activiy (Diameter of Inhibition Zone, mm)		Cytotoxicity Assay (IC_50_, μg/ mL)	
	*B.cereus*	*P.aeruginosa*	MRC5	Caco-2
F1	0.0	8.9 ± 0.3	No inhibition	35 *
F6	14.2 ± 0.6	7.3 ± 0.2	420 **	130 ***
F7	14.2 ± 0.7	7.3 ± 0.1	No inhibition	65 **
F8	13.5 ± 0.4	7.4 ± 0.2	No inhibition	70 **
F9	25.4 ± 0.5	7.5 ± 0.2	No inhibition	85 ***

Significance levels: * *p* < 0.05, ** *p* <0.01, *** *p* <0.001.

## Data Availability

The original contributions presented in this study are included in the article. Further inquiries can be directed to the corresponding author(s).

## Abbreviations

The following abbreviations are used in this manuscript:

SLs	Sophorolipids
RBD PO	Bleached and deodorized palm olein
RBD PKO	Bleached and deodorized palm kernel olein
RBD CO	Bleached and deodorized coconut olein
FAME	Fatty acid methyl ester
MCT	Medium-chain triglyceride
IFT	Interfacial tension
CMC	Critical micelle concentration
